# Derivation of two iPSC lines (KAIMRCi004-A, KAIMRCi004-B) from a Saudi patient with Biotin-Thiamine-responsive Basal Ganglia Disease (BTBGD) carrying homozygous pathogenic missense variant in the *SCL19A3* gene

**DOI:** 10.1007/s13577-024-01097-4

**Published:** 2024-07-09

**Authors:** Maryam Alowaysi, Moayad Baadhaim, Mohammad Al-Shehri, Hajar Alzahrani, Amani Badkok, Hanouf Attas, Samer Zakri, Seham Alameer, Dalal Malibari, Manal Hosawi, Mustafa Daghestani, Khalid Al-Ghamdi, Mohammed muharraq, Asima Zia, Jesper Tegne, Majid Alfadhel, Doaa Aboalola, Khaled Alsayegh

**Affiliations:** 1 King Abdullah International Medical Research Center (KAIMRC), King Saud Bin Abdulaziz University for Health Sciences, King Abdulaziz Medical City, Jeddah, Saudi Arabia; 2 Clinical Biomedical Genetics, Ministry of the National Guard Health Affairs, Jeddah, Saudi Arabia; 3 Department of Pathology and Laboratory Medicine, Ministry of the National Guard Health Affairs, Jeddah, Saudi Arabia; 4 Forensic Laboratories, Criminal Evidence Department, Jeddah, Saudi Arabia; 5 Biological and Environmental Science and Engineering Division, King Abdullah University of Science and Technology (KAUST), Thuwal, Saudi Arabia; 6 Computer, Electrical and Mathematical Sciences and Engineering Division, King Abdullah University of Science and Technology (KAUST), Thuwal, Saudi Arabia; 7 Department of Genetics and Precision Medicine, King Abdullah Specialized Children Hospital, King Abdulaziz Medical City, Ministry of National Guard Health Affairs, Riyadh, Saudi Arabia; 8 Department of Medical Genomics Research, King Abdullah International Medical Research Center, Ministry of National Guard Health Affairs, King Saud Bin Abdulaziz University for Health Sciences, Riyadh, Saudi Arabia

**Keywords:** iPSC, Biotin-thiamine-responsive basal ganglia disease, BTBGD, Encephalopathy, *SLC19A3* variant, Saudi Arabia

## Abstract

The neurometabolic disorder known as biotin-thiamine-responsive basal ganglia disease (BTBGD) is a rare autosomal recessive condition linked to bi-allelic pathogenic mutations in the *SLC19A3* gene. BTBGD is characterized by progressive encephalopathy, confusion, seizures, dysarthria, dystonia, and severe disabilities. Diagnosis is difficult due to the disease’s rare nature and diverse clinical characteristics. The primary treatment for BTBGD at this time is thiamine and biotin supplementation, while its long-term effectiveness is still being investigated. In this study, we have generated two clones of induced pluripotent stem cells (iPSCs) from a 10-year-old female BTBGD patient carrying a homozygous mutation for the pathogenic variant in exon 5 of the *SLC19A3* gene, c.1264A > G (p.Thr422Ala). We have confirmed the pluripotency of the generated iPS lines and successfully differentiated them to neural progenitors. Because our understanding of genotype–phenotype correlations in BTBGD is limited, the establishment of BTBGD-iPSC lines with a homozygous SLC19A3 mutation provides a valuable cellular model to explore the molecular mechanisms underlying SLC19A3-associated cellular dysfunction. This model holds potential for advancing the development of novel therapeutic strategies.

**Supplementary Information:**

The online version contains supplementary material available at 10.1007/s13577-024-01097-4.

## Introduction

Biotin-thiamine-responsive basal ganglia disease (BTBGD) (MIM: 6,07,483) is a rare autosomal recessive disorder that affects the basal ganglia characterized by neurological dysfunction [1]. Also known as thiamine transporter 2 deficiency or thiamine metabolism dysfunction syndrome 2 (THMD2), BTBGD results from mutations in the solute carrier family 19, member 3 (*SLC19A3*, MIM: 6,06,152) gene, which encodes for the thiamine transporter 2 (THTR-2). THTR-2 plays a crucial role in transporting thiamine (vitamin B1) into cells, particularly within the central nervous system [2]. Mutations in the *SLC19A3* gene may result in a reduced ability to transport thiamine into cells, which can lead to decreased absorption of the vitamin and neurological dysfunction [3]. Globally, it is estimated that BTBGD prevalence at birth is 1 in 2,15,000, and a carrier frequency of 1 in 232 in the general population has been estimated for all BTBGD phenotypes. Notably, the Middle Eastern populations exhibit a disproportionate burden of this disease, with a carrier frequency estimated at 1 in 500 individuals in Saudi newborns [4]. However, the overall low prevalence of the condition may be attributed to a combination of misdiagnosis and underdiagnosis, implying that with enhanced diagnostic precision and reporting, the detection and documentation of additional cases are likely [1].

According to *Maney *et al*.* (2023) and *Sharma & Saini *et al*.* (2021), biotin-thiamine-responsive basal ganglia disease presents in infancy (infantile BTBGD), childhood (early childhood encephalopathy) or adulthood (late-onset Wernicke-like encephalopathy) [1, 5]. First, the early infantile BTBGD that presents within the first few months of life, often during the neonatal period. Movement abnormalities, developmental regression, seizures, altered mental status, feeding difficulties, and bilateral basal ganglia lesions characterize this phase. Next, early childhood encephalopathy normally presents between the ages of 3 and 10. It is characterized by episodic encephalopathy triggered by fever, metabolic stress, trauma, vaccination, or extensive exercise. Other signs include neuro-regression, recurrent seizures (even status epilepticus), spasticity, severe extrapyramidal involvement, gait abnormalities, behavioral problems, and bilateral affection of the corpus striatum and cerebral cortex, with or without brain stem involvement. Finally, late-onset Wernicke-like encephalopathy presents with mental confusion, ataxia, ophthalmoplegia, cognitive impairment, and gait disturbances. This phase, predominant in young adults in their twenties, is associated with compound heterozygous variations in the *SLC19A3* gene [6].

The primary treatment for BTBGD is thiamine and biotin supplementation, which attempts to enhance thiamine’s transportation into the brain [5]. Because biotin treatment has a positive effect, it is important to diagnose this illness as soon as possible to enable adequate management and prevent needless tests and treatments. To completely comprehend the pathophysiology and management of this uncommon ailment, more investigation is necessary. Biotin-thiamine-responsive basal ganglia disease could be fatal if left untreated, emphasizing the need for early diagnosis and proper management of the medical condition [2]. The recommended methods for diagnosis are magnetic resonance imaging for brain diagnostics; laboratory tests and genetic testing are used to validate imaging [5].

With the advent of stem cell research and organoid technology [7], it has become possible to create in vitro models of diseases like BTBGD. An in-depth understanding of the pathophysiological mechanisms underlying BTBGD is crucial for the improvement of management strategies. The generation of induced pluripotent stem cells (iPSCs) from patients diagnosed with BTBGD provides a promising avenue for cellular-level disease investigation. Using iPSCs, brain organoid technology could be employed to simulate disease pathology and screen potential therapeutic agents [8].

In this study, we derived iPSCs (two clones) by reprogramming peripheral blood mononuclear cells (PBMCs) of a Saudi patient with BTBGD carrying a homozygous mutation in the *SLC19A3* gene c.1264A > G (p.Thr422Ala). The pluripotency of these iPS lines and their ability to generate neural progenitors has been confirmed. These iPSCs make an invaluable tool for elucidating disease mechanisms and developing patient-specific therapies, including cell replacement strategies and personalized pharmacological interventions [8].

## Materials and Methods

### Patient recruitment and ethical approval

This study was approved by the institutional review board (IRB) and research ethics committee of KAIMRC (NRJ22J/005/01) and (NRJ22/060/03). The patient is a 10-year-old female diagnosed with Biotin-Thiamine-Responsive Basal Ganglia disease-carrying the known familial pathogenic variant in the exon 5 of the *SLC19A3* gene, c.1264A > G (p.Thr422Ala) in a homozygous state. The informed consent forms (ICFs) were used to obtain and process the patient’s samples with the approval of the patient’s parents.

### PBMCs isolation and enrichment of erythroid progenitors

EDTA-containing blood collection tubes were used to collect peripheral blood from the patient and process it with the RosetteSep™ Human Progenitor Cell Basic Pre-Enrichment antibody cocktail (Stem Cell Technologies Catalog#15,226). Following PBMC separation and isolation, 1 million cells were cultured for 8 days in StemSpan™ SFEM II medium with 1X StemSpan™ Erythroid Expansion Supplement (Stem Cell Technologies Catalog #02692).

### Erythroid progenitor cells transfection

EPCs reprogramming was performed using the Epi5 Reprogramming Kit (Thermofisher Catalog#A15960). Three pulses at 1600 V, each lasting 10 ms, were used to transfect the cells with 1 μg of each episomal vector (Neon Transfection System, Thermofisher). Subsequently, iPS colonies were picked and cultured using mTeSR™ Plus medium and Geltrex™ LDEV-Free Reduced Growth Factor Basement Membrane Matrix (Thermofisher Catalog# A1413201) at 37 °C with 5% CO_2_ and 20% O_2_.

### Immunocytochemistry

The initial fixation of the cells was done for a period of 15 min in 4% (w/v) paraformaldehyde. This was followed by another 10-min wash with PBS solution consisting of 0.1% (v/v) Triton X-100. Subsequently, the cells were blocked with a PBS solution containing 1% gelatin for a period of 45 min. The cells were then probed overnight at 4 °C with the primary antibodies and for an hour at room temperature with the appropriate secondary antibodies. The nuclei were stained with DAPI nuclear staining at 1 μg /mL. We observed the staining using a 20X objective on a Zeiss LSM 880 Airyscan confocal laser scanning microscope.

### Quantitative reverse transcription PCR (RT-qPCR)

The total RNA was extracted following the manufacturer’s instructions using the RNeasy Mini Kit. Subsequently, 500 ng of RNA was reverse-transcribed using High-Capacity cDNA Reverse Transcription Kit (Applied Biosystems Catalog#4,368,814). Real-time qPCR reactions were performed on the QuantStudio 7 Flex Real-Time PCR System (Thermofisher scientific) using the *Power*SYBR Green Master Mix (ThermoFisher scientific Catalog#4,367,659).

### In vitro differentiation

To develop the three germ layers derivatives from the iPSCs, STEMdiff™ Trilineage Differentiation Kit was used (Stem Cell Technologies Catalog #05230). The induction of ectodermal and endodermal differentiation was performed by seeding 2 × 10^5^ cells/cm^2^ and feeding daily with lineage-specific medium for 6 and 4 days, respectively. The mesodermal lineage required 5 × 10^4^ cells/cm^2^ and medium replacement for 4 days. The differentiation ability into the three germ layers—mesoderm, endoderm, and ectoderm—was evaluated by RT-qPCR and Flow cytometry analysis (Table 1).

**Table 1:** List of antibodies and primers used in this study

Antibodies and stains used for immunocytochemistry/flow cytometry			
	Antibody	Dilution	Company Cat # and RRID
Pluripotency markers	Rabbit anti-OCT4	1:100	Abcam Cat# ab200834 RRID# AB_2924374
	Goat anti-NANOG	1:100	Abcam Cat# ab109250 RRID# AB_10863442
	Goat anti-SOX2	1:100	Thermofisher Cat# MA1-014 RRID# AB_2536667
Trilineage markers	Mouse anti-Nestin	1:25	Stem cell technologies Cat#60091AD
	Mouse anti-SOX17	1:50	Thermofisher Cat# MA5-24,885 RRID# AB_2725396
	Mouse anti- Brachyury	1:50	R&D systems Cat# IC2085G
NPCs markers	Rabbit anti-ASCL1	1:100	Thermofisher Cat# PA5-77,868 RRDI# AB_2735483
	Mouse anti-TH	1:50	Thermofisher Cat# MA1-24,654 RRDI# AB_795666
	Mouse anti-Nestin	1:25	Stem cell technologies Cat#60091AD
Secondary antibody	Goat anti-Rabbit Secondary Antibody, Alexa Fluor 488	IF 1:200 Flow Cyt 1:2000	Abcam Cat#: ab150077 RRID# AB_2630356
	Goat anti-Mouse Secondary Antibody, Alexa Fluor 488	IF 1:200 Flow Cyt 1:100	Thermofisher Cat#35,502 RRID# AB_844397

Primers and oligonucleotides used in this study		
	Target	Forward/Reverse primer (5′-3′)
Differentiation markers RT-qPCR	BRACHYURY or TBXT	TAAGGTGGATCTTCAGGTAGC CATCTCATTGGTGAGCTCCCT
	CDX2	GACGTGAGCATGTACCCTAGC GCGTAGCCATTCCAGTCCT
	NESTIN	CTGCTACCCTTGAGACACCTG GGGCTCTGATCTCTGCATCTAC
	PAX6	TGGGCAGGTATTACGAGACTG ACTCCCGCTTATACTGGGCTA
	SOX17	GCATTCTGGAATGAGCCTACT GGGCAGGTCAAGCTTATGAT
	GATA4	CGACACCCCAATCTCGATATG GTTGCACAGATAGTGACCCGT
House-keeping genes (qPCR)	GAPDH	GGAGCGAGATCCCTCCAAAAT GGCTGTTGTCATACTTCTCATGG
mutation analysis	SLC19A3	TCTCTCTCTCTCTTTGCTG ACTTCTTACCTGCCTTATCC
EBNA-1	pEP4-SF2-oriP pEP4-SR2-oriP	ATC GTC AAA GCT GCA CAC AG CCC AGG AGT CCC AGT AGT CA

### Neural progenitor cells (NPCs) differentiation

Using the STEMdiff™ SMADi Neural Induction Kit (Stem Cell Technologies Catalog #08581), BTBGD-iPSCs were differentiated into NPCs following the manufacturer’s protocol. Briefly, iPSCs were generated using the monolayer culture protocol and seeded at 2 × 10^5^ cells/cm^2^ on Matrigel-coated tissue culture plate (Corning). NPCs were passaged three times and fed with NPCs STEMdiff™ Neural Induction Medium + SMADi for 21 days. NPCs were further expanded at 1.25 × 10^5^ cells/cm^2^ in STEMdiff™ Neural Progenitor Medium for 7 days.

### Flow cytometry analysis

Permeabilization and staining for intracellular markers were performed using the BD IntraSure™ Kit (BD Biosciences Catalog# 641,778). Reagent A was used to fix 4 × 10^5^ cells for 10 min. Upon diluting primary antibodies with reagent B, cells were incubated for 30 min. PBS was used to dilute the secondary antibodies before they were incubated at room temperature for 30 min. Using BD FACS ARIA cell sorter, FACS samples were analyzed. A comparison of stained vs unstained cells was performed to determine the percentage of FITC-positive cells.

### Karyotype analysis

iPSCs were treated for 15 min with KaryoMAX™ Colcemid™, 0.3 μg/mL, and then dissociated by TrypLE after treatment. A hypotonic solution of 75 mM potassium chloride was used to incubate the cells for 20 min at 37 °C, and then iPSCs were fixed in methanol and glacial acetic acid in a 3:1 solution. Pathology and laboratory medicine (Ministry of the National Guard—Health Affairs) performed the karyotyping on at least 20 metaphases.

### Plasmids screening

DNA was extracted according to the manufacturer’s instructions using the All Prep DNA/RNA/Protein Mini Kit (Qiagen Catalog# 80,004). As part of the PCR procedure, EBNA-1 primers were used to identify the five episomal plasmids (expected size 666 bp) (Thermo Fisher Scientific Catalog # A15560).

### Short tandem repeat (STR)

In this study, fifteen STR loci and amelogenin were amplified using the AmpFLSTR™ Identifiler™ Plus PCR Amplification Kit (Applied Biosystems Catalog#4,427,368). The samples were amplified using the kit then run on 3500 Genetic Analyzer to determine the PCR amplicons. In order to gather and evaluate the data, the GeneMapper ID-X Software, version 1.4, was used to collect the results.

### Mycoplasma detection

Mycoplasma contamination was assessed using LookOut® Mycoplasma qPCR Detection (SIGMA).

### Statistical analysis

In this study, RT-PCR data were expressed as mean ± standard deviation (SD). The significance of the analysis was evaluated using the Student’s *t*-test (unpaired; two-tailed). To correct for multiple comparisons, a Bonferroni correction was applied to the *p*-value.

## Results

### A description of clinical data and a mutation analysis

**Fig. 1:**
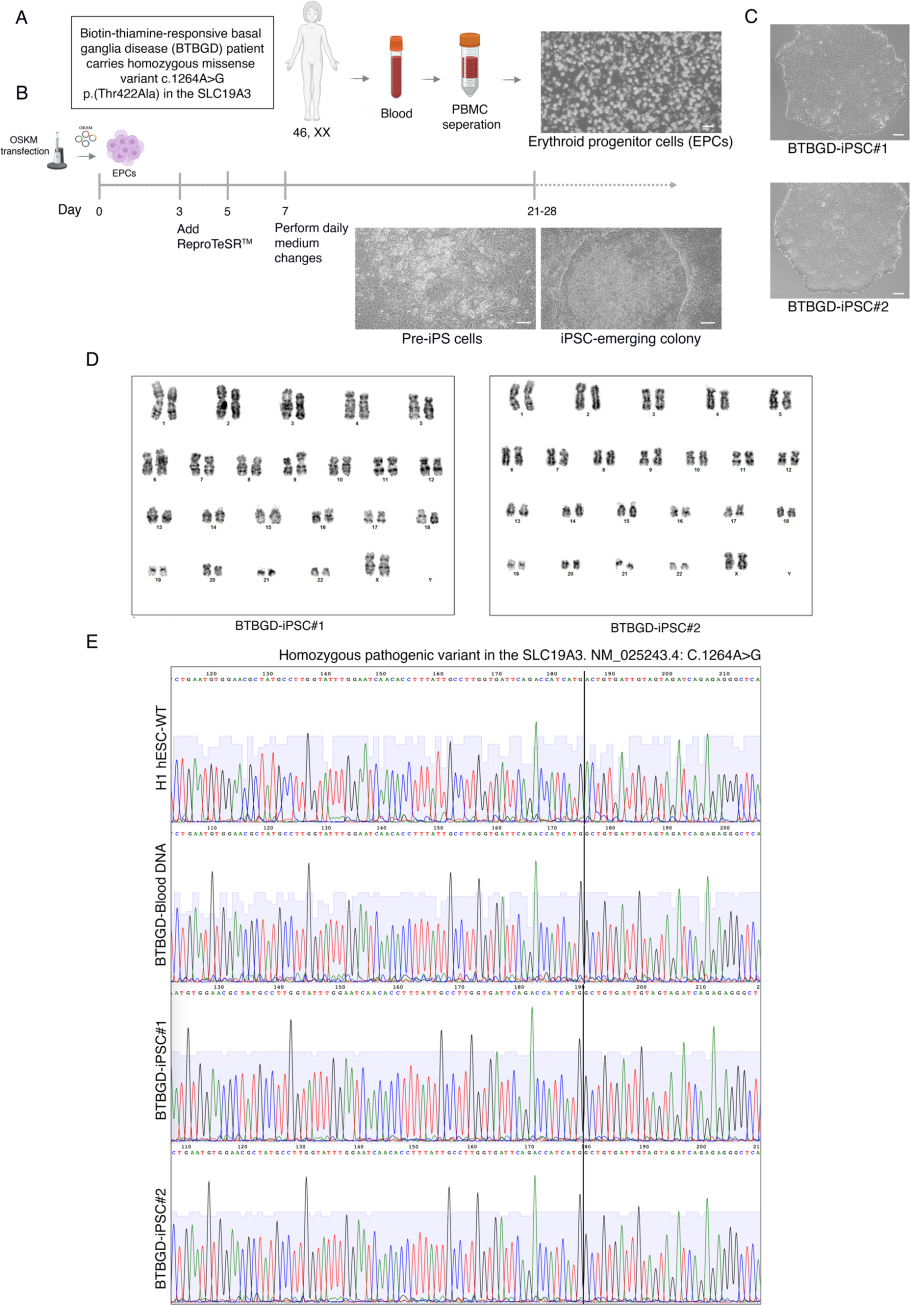
Cell reprogramming and derivation of BTBGD-iPSCs using the following methods. **A** A sample of 10 ml peripheral blood from a BTBGD patient was expanded for 8 days to enrich for erythroid progenitor cells (EPCs). **B** Schematic representation of ReproteSR™ and episomal reprogramming. Phase-contrast images show the transition between mesenchymal and epithelial cells, as well as the emergence of colonies during reprogramming (days 11 to 28). **C** BTBGD-iPSC clones are tightly packed clones with well-defined borders. Scale bar = 100 μm. **D** Typical G-banded karyotype tests show that the karyotypes of BTBGD-iPSCs have normal chromosomal content 46, XX. **E**. SLC19A3 mutations in the H1 hESC-wild type, patient’s peripheral blood cells, and the BTBGD-iPSC lines are shown in the electropherogram record of Sanger sequencing

The 10-year-old female patient presented with a history of seizures, subacute encephalopathy, developmental delay, and sensorineural hearing loss. Molecular genetic analysis by whole exome sequencing (WES) of the patient’s blood sample identified a homozygous pathogenic variant in the *SLC19A3*, c.1264A > G (NP_079519.1:p.Thr422Ala). This mutation leads to an amino acid exchange in exon 5 (NM_025243.4) and has been previously described as disease-causing for biotin-thiamine-responsive basal ganglia disease by *Alfadhel *et al*.*, 2013 [6]. This variant was confirmed in the patient’s peripheral blood cells as well as in the derived iPSC lines by Sanger sequencing (Fig. 1E).

### The derivation and establishment of BTBGD-iPSC lines

An in-person interview was conducted and signed informed consent was obtained from the donor’s parent. Erythroid progenitor cells (EPCs) were isolated and enriched from a 10 ml peripheral blood sample and cultured for 8 days (Fig. 1A). Using a non-integrative and virus-free reprogramming technique, as previously described [9, 10], two BTBGD-iPSC clones were created. Briefly, episomal vectors encoding OCT4, SOX2, KLF4, L-MYC, LIN28A, dominant-negative form of TP53, and EBNA-1 were delivered to EPCs by electroporation (Fig. 1B). Several ESC-like colonies displaying typical ESC morphological characteristics (including distinct borders, bright centers, tightly packed cells, and a high nucleus-to-cytoplasm ratio) were identified approximately 20 days post-transfection (Fig. 1C). The derived iPSC lines were manually picked, expanded in feeder-free conditions, and cryopreserved at KAIMRC facility.

A female normal chromosomal content was confirmed by G-banding analysis of the generated BTBGD-iPSCs (Fig. 1D.) The matched identities of the isolated iPS lines and the donor PBMCs have been validated by short tandem repeats (STR) assay (Fig. S1B). Furthermore, mycoplasma testing has indicated that the generated iPSC are free from mycoplasma contamination (Fig. S1C).

### Characterization of self-renewal and potency properties

**Fig. 2:**
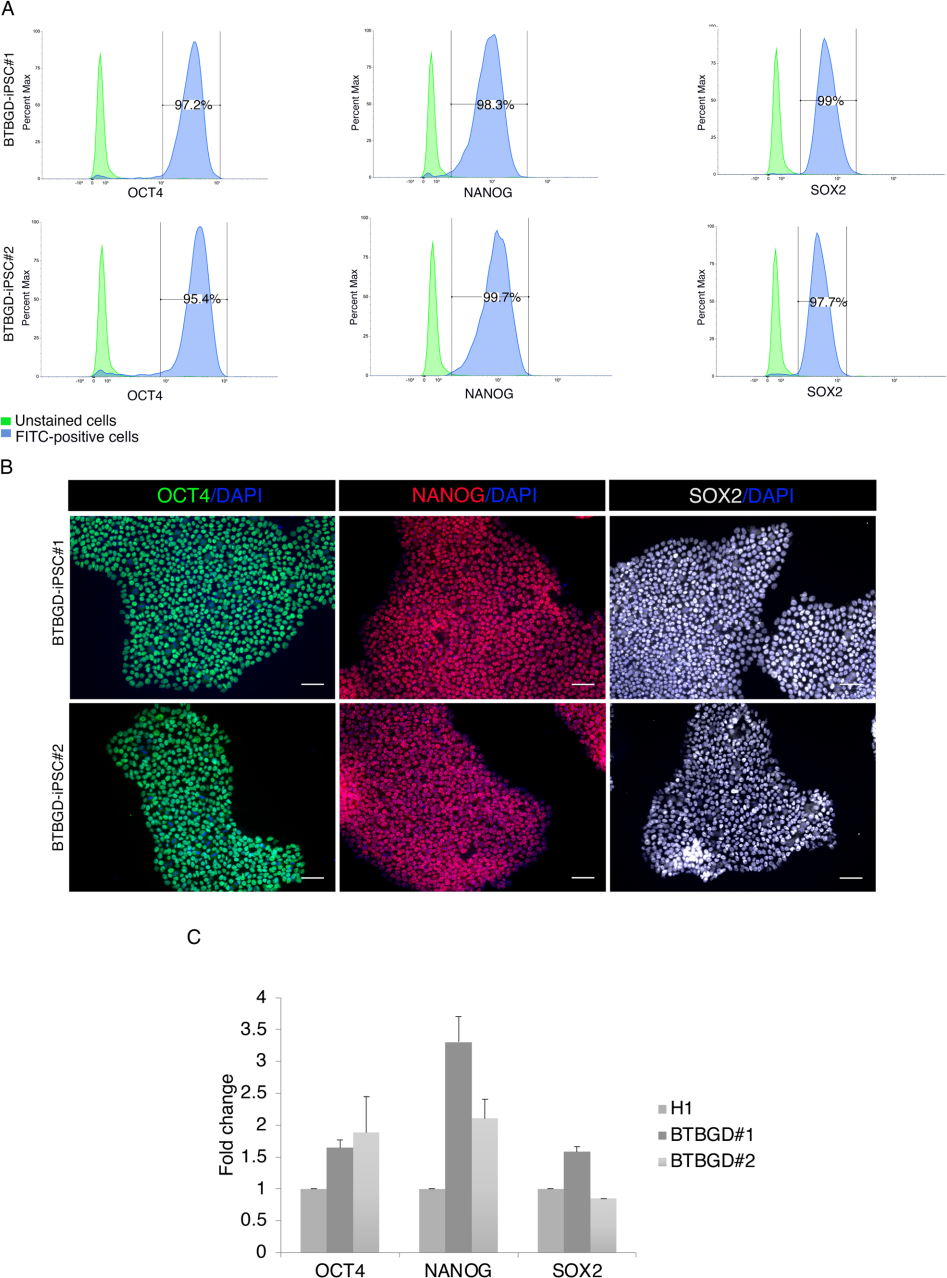
Analysis of the pluripotency in the generated BTBGD-iPSCs. **A** Flow cytometry histograms of OCT4, NANOG, and SOX2 in BTBGD-iPSCs. **B** immunofluorescence staining of the pluripotency markers OCT4 (green), NANOG (red), and SOX2 (yellow), Nuclei were stained with DAPI (blue). Scale bar = 50 μm. **C** mRNA expression levels of pluripotency markers for the indicated iPSC lines are presented as a fold change in comparison to H1 hESC. Bars represent the median ± std of three biological replicates for each sample

Manually picked clones were passaged and analyzed for the presence of episomal plasmids at every passage. Complete absence of reprogramming plasmids became evident at passage twelve (Fig. S1A). Consequently, we assessed the expression of pluripotency markers (OCT4, NANOG, and SOX2) using flow cytometry, immunofluorescence, and real-time PCR (RT-qPCR). According to flow cytometry histograms, more than 95% of cells expressed OCT4, 98% expressed NANOG, and 97% were positive for SOX2 (Fig. 2A). Furthermore, immunofluorescence staining showed positive expression of stemness markers in the derived iPS lines (Fig. 2B). Using RT-qPCR, we found that in comparison to H1 hESCs, the expression of *OCT4, NANOG*, and *SOX2* mRNA was significantly upregulated (Fig. 2C).

**Fig. 3:**
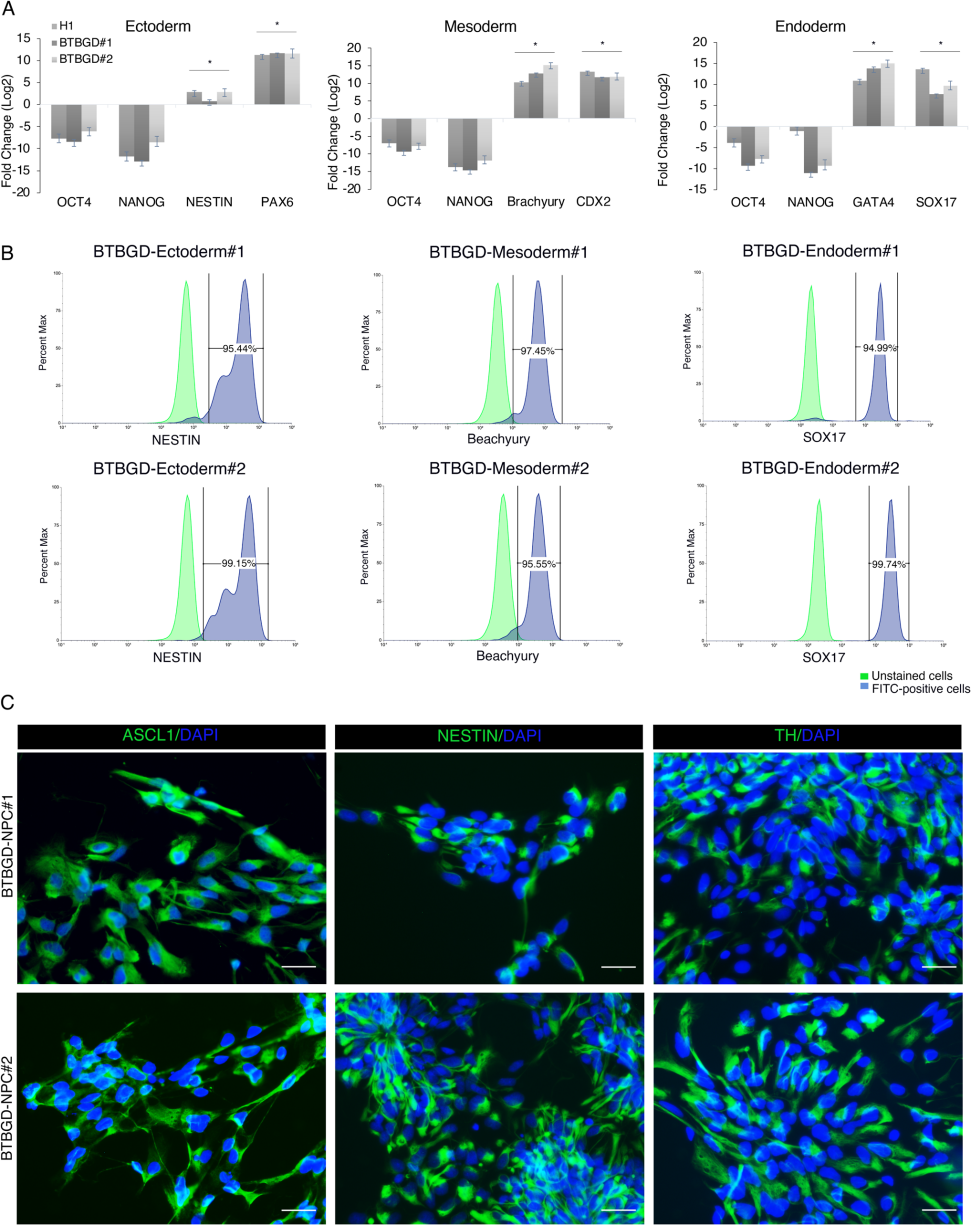
Assessing Trilineage and Neural Progenitor Cells (NPCs) differentiation. **A** mRNA expression levels of the lineage-specific markers for the three germ layers—ectoderm (NESTIN and PAX6), mesoderm (CDX2 and Brachyury), and endoderm (GATA4 and SOX17)—presented as fold changes in comparison to undifferentiated cells. Bars are median ± std of 3 biological replicates for each sample. Student’s *t*-tests, *p < 0.05. **B** Flow cytometry histograms of NESTIN, Brachyury, and SOX17 in the tissues derived from the three embryonic germ layers. **C** immunofluorescence staining of the NPC markers ASCL1, NESTIN, and TH (Green) in generated BTBGD-NPCs. Nuclei were stained with DAPI (blue). Scale bar = 20 μm

The capacity of the generated iPSCs to differentiate into the three germ layers—mesoderm, endoderm, and ectoderm—was assessed through direct in vitro differentiation. Upregulation of germ layer-specific markers and downregulation of pluripotency markers *OCT4* and *NANOG* were observed across all lineages. Ectodermal differentiation has been proven by the positive expression of the central nervous system neural progenitor markers *PAX6* and *NESTIN*. The capacity of mesodermal commitment was assessed by directed in vitro differentiation and was demonstrated by an increase in the expression of *CDX2*, a caudal-type homeobox protein 2, and *Brachyury*, a member of the T-box family. The upregulation of the endodermal markers zinc-finger transcription factor *GATA4* and the SRY-Box transcription factor 17 (*SOX17*) has been validated in our BTBGD-iPSC lines and H1 hESC-positive controls (Fig. 3A). Furthermore, following direct in vitro differentiation, the resulting cells were also evaluated for germline markers expression using flow cytometry. More than 95% of the cells were positive for NESTIN, Brachyury, and SOX17, in ectodermal, mesodermal, and endodermal differentiation respectively (Fig. 3B).

Following pluripotency verification, the generated BTBGD-iPSC lines have been registered in the Human Pluripotent Stem Cell Registry https://hpscreg.eu/user/cellline/edit/KAIMRCi004-Ahttps://hpscreg.eu/user/cellline/edit/KAIMRCi004-B.

### Efficient generation of neural progenitor cells from BTBGD-iPSCs

Neurological dysfunction is one of the main pathological characteristics of BTBGD patients [1]. Consequently, research on human midbrain neurons produced from hiPSCs may provide insight into the pathogenic pathways underlying BTBGD. To evaluate BTBGD-iPSC line’s capacity to produce neural progenitor cells (NPCs), we generated CNS-type NPCs using Dual SMAD inhibition (SMADi) direct differentiation for 28 days in a monolayer culture format. Around 90% of the area of the adhered cells was filled with NPCs positive for the achaete-scute family bHLH transcription factor 1 (ASCL1) that contributes to the development of olfactory and autonomic neurons as well as to the commitment and differentiation of neurons. We further observed a positive expression of the neuroepithelial stem cell protein (NESTIN), and tyrosine hydroxylase (TH), which is involved in the synthesis of catecholamine neurotransmitters dopamine (Fig. 3C). These data revealed a highly efficient creation of pure CNS-type NPCs that will be further differentiated into downstream cell types such as neurons.

## Discussion

The creation of iPSCs and the revolutionary discovery of cellular reprogramming have been widely used in the past 10 years to simulate diseases in vitro and offer the potential for scientific research and regenerative therapies [11, 12]. iPSCs and embryonic stem cells have many features of self-renewal, gene expression, and the ability to develop into almost any type of body cell [10]. NANOG, OCT3/4, SOX2, KLF4, c-MYC, and LIN28 are pluripotency transcription factors that regulate the expression of stemness and repress somatic genes [13–21]. Even though iPSCs can be generated from a variety of somatic cell sources, we opted for EPCs for their tendency to be devoid of genomic DNA mutations or chromosomal abnormalities [9, 10]. We found that 69% of EPCs were positive for CD71^+^CD235a^+^ erythroid cell surface markers after 8 days of expansion in erythroid expansion media [9]. To generate integration-free iPSCs, non-viral and non-integrating episomal plasmid-based reprogramming method was applied in this study [9, 10]. Based on the Epstein–Barr Nuclear Antigen-1, vectors incorporating oriP and EBNA-1 have demonstrated the capacity to generate iPSCs successfully with a single transfection [9, 10].

Homozygous presence of the familial pathogenic variant c.1264A > G (p.Thr422Ala) in the *SLC19A3* gene has been previously delineated as causative for biotin-thiamine-responsive basal ganglia disease (BTBGD) by Alfadhel et al., 2013 [6]. BTBGD represents a remarkably rare genetic condition, the diagnosis of which is complicated by its nonspecific clinical manifestations. These typically include seizures and encephalopathy, compounded by a broad spectrum of imaging differentials, including cortical T2-hyperintensities and bilateral involvement of the basal ganglia [22]. It has also been established that the prognosis of BTBGD is significantly compromised by the delayed administration of biotin and thiamine, underscoring the necessity for timely intervention [6, 23].

The pathogenesis of BTBGD caused by SLC19A3 deficiency remains unclear. Therefore, the derivation of BTBGD-iPSC lines carrying *SLC19A3* mutation constitutes a suitable research model to study genotype–phenotype correlations. Furthermore, differentiation of BTBGD-iPSCs toward disease-relevant lineages, such as neuronal subtypes and midbrain organoids, would serve as a powerful cellular platforms to determine the impact of SLC19A3 deficiency and the underlying disease mechanism in BTBGD. CRISPR/Cas9-mediated knock-in of *SLC19A3*, coupled with in vitro disease modeling using midbrain organoids and gene expression profiling could be utilized to further our understanding of BTBGD pathogenesis, thus allowing for the discovery of more efficient therapeutic agents through drug screening.

### Supplementary Information

Below is the link to the electronic supplementary material.

Supplementary file1 Fig. 1S: A. Endpoint PCR demonstrated the absence of the five episomal plasmids in the BTBGD-iPSCs. B. Short Tandem Repeat (STR) profiling guaranteed the genetic identity between the established iPSC lines and the donor PBMCs. C. RT-qPCR showing negative mycoplasma test in BTBGD-iPSC lines (TIF 180819 KB)

## Data Availability

The data that support the findings of this study are openly available. All characterization data related to this study can be accessed upon reasonable request. Requests for access to this data should be directed to Dr. Khaled Alsayegh, alsayeghk@kaimrc.edu.sa and/or Dr. Doaa Aboalola, aboalolad@kaimrc.edu.sa.
